# Signal enhancement in supercritical fluid chromatography‐diode‐array detection with multiple injection

**DOI:** 10.1002/jssc.201900614

**Published:** 2019-11-19

**Authors:** Mingzhe Sun, Charlotta Turner, Margareta Sandahl

**Affiliations:** ^1^ Department of Chemistry Centre for Analysis and Synthesis Lund University Lund Sweden

**Keywords:** large injection volume, multiple injection, signal enhancement, supercritical fluid chromatography

## Abstract

To circumvent the detrimental effects of large‐volume injection with fixed‐loop injector in modern supercritical fluid chromatography, the feasibility of performing multiple injection was investigated. By accumulating analytes from a certain number of continual small‐volume injections, compounds can be concentrated on the column head, and this leads to signal enhancement compared with a single injection. The signal to noise enhancement of different compounds appeared to be associated with their retention on different stationary phases and with type of sample diluent. The diethylamine column gave the best signal to noise enhancement when acetonitrile was used as sample diluent and the 2‐picolylamine column showed the best overall performance with water as the sample diluent. The advantage of multiple injection over one‐time large‐volume injection was proven with sulfanilamide, with both acetonitrile and water as sample diluents. The multiple injection approach exhibited comparable within‐ and between‐day precision of retention time and peak area with those of single injections. The potential of the multiple injection approach was demonstrated in the analysis of sulfanilamide‐spiked honey extract and diclofenac‐spiked ground water sample. The limitations of this approach were also discussed.

**Article Related Abbreviations**
1‐AA: 1‐aminoanthrocene
2‐PIC: 2‐picolylamine
ACN: acetonitrile
DEA: diethylamine
NSAID: nonsteroidal anti‐inflammatory drugs

## 1 INTRODUCTION

As a good complement to traditional reversed‐phase liquid chromatography, SFC has drawn more and more attention in the past decade 1. Compared with reversed‐phase liquid chromatography, SFC offers a wider range of stationary phase chemistry selectivities with a lower consumption of organic solvent 2. Major application fields of SFC include pharmaceutical analysis, biological sample analysis, natural and food product analysis 3, 4, 5, 6. Revolutionary development in modern SFC instrumentation has also brought dramatic improvement in noise reduction, which opens up the possibility for trace analysis of environmental and biological samples 7, 8, 9, 10. For such analysis, SFC methods with enhanced analyte detectability are in most cases needed, enabling for lower detection limits.

Regardless of the type of detector used after column separation, one simple way to boost the detectability in any LC method is to increase the injection volume. As the compounds can be temporarily trapped on the column head, analyte bands can become more concentrated with larger volume of sample injected 11. However, for a specific sample diluent, the injection volume can only be increased to a certain amount before it brings unacceptable negative effects on chromatography performances 12, 13.

The trade‐off between detectability and peak resolution by selection of sample diluent and injection volume is especially crucial in SFC 14, 15, 16, 17. Compared with HPLC, SFC demands special designs of injection. The classical variable‐loop auto‐sampler is not appropriate for SFC use as the metering device can be cavitated by the expansion of the mobile phase 18. Instead, most of the modern SFC systems use fixed‐loop injection to isolate the metering device from the loop and rotor. Even though extra work is demanded, different injection volume is possible by installing injection loops of different volumes. However, the injection volume is set to below 10 µL in most analytical SFC applications reported so far 6, 19, 20, which is the consequence of three major factors: strong solvent effect, viscous fingering, and pressure consideration. The rule‐of‐thumb of dissolving the sample in a solvent that is as similar as or even weaker than the mobile phase starting composition is clearly not applicable in SFC, since compressed CO_2_ is not possible to be used as sample diluent. The sample diluent in SFC often consists of a single or a mixture of organic solvents, which unavoidably leads to a strong solvent effect, especially for poorly retained compounds 14, 16. Deformation of the injection plug can also take place due to the large viscosity difference between the injection plug and the mobile phase 21. Although being among the least suitable SFC sample diluent, water has been proven to offer beneficial effects in several studies, especially when acetonitrile is used to dilute the aqueous sample 22, 23, 24, 25. One particular obstacle associated with water‐containing injection is that it can cause a huge pressure spike right after the injection, possibly due to the pumping of a water plug through the column at high SFC flow rates 26, 27.

**Figure 1:**
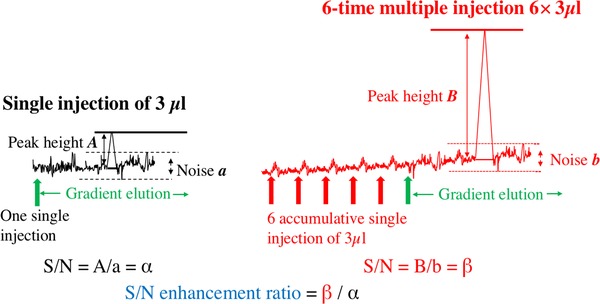
Calculation of the S/N enhancement ratio for a multiple injection analysis

In order to decrease the negative effects of large volume injection in SFC and strive for higher detectability without sacrificing the injection flexibility, this work evaluated the possibility of performing multiple accumulative injections in modern analytical packed‐column SFC using sub‐2 µm particles. The multiple injection approach was briefly introduced to enhance detectability in the early days of SFC when capillary columns were used 28. When the mobile phase starts at a composition that has very limited elution strength, some analytes from multiple injections can be effectively trapped and accumulated on the head of the column. In this study, a number of rapid small‐volume injections were made continually with low‐elution strength 100% CO_2_ as the mobile phase. This was then followed by a final one‐time gradient elution with co‐solvent and separation of the sample. A schematic elucidation of the approach is shown in Supporting Information Figure S1. The gain in detectability can be measured by the S/N enhancement ratio of the analyte peak. Figure 1 is a simplified drawing that shows how the S/N enhancement ratio is calculated for a multiple injection analysis. In this work, the signal enhancement arising from different number of injections (up to 20) was first investigated on different stationary phases with compounds of a wide variety of physiochemical properties dissolved in both acetonitrile (ACN) and water (Supporting Information Table S1). The multiple injection approach was then applied for the analysis of sulfanilamide in spiked honey extract and diclofenac in spiked ground water as proofs of concept. One important fact that is important to note is that with increasing proportion of methanol in the mobile phase, the critical points of the mobile phase change drastically. Consequently, the separations in the latter period are technically in the liquid state. Even though the change from supercritical to liquid state does not cause significant change in mobile phase properties, as long as the fluid is in a single‐phase, the inaccuracy of definition should still be pointed out for better clearance 1.

## 2 MATERIALS AND METHODS

### 2.1 Reagents

Caffeine was purchased from Merck (Darmstadt, Germany). Fluoranthene, ibuprofen, p‐hydroxybenzaldehyde, p‐coumaric acid, 3‐methoxycinnamic acid, ferulic acid, sulfanilamide, and diclofenac were purchased from Sigma Chemical (St. Louis, Missouri, USA). Carbon dioxide of 5.3 purity grade was purchased from Linde (Guildford, UK). Methanol and acetonitrile (HPLC grade) used were purchased from VWR (Radnor, Pennsylvania, United States). Milli‐Q water was used in all experiments. The compound concentration in all standard solutions was 50 µg/mL dissolved in both acetonitrile and water (fluoranthene and ibuprofen in water were not studied because of poor solubility). Approximately 3 mL honey purchased from a local market was extracted with 3 mL acetonitrile and spiked with sulfanilamide to 500 ng/mL. Ground water sample was collected from a small lake near the lab, filtrated and then spiked with diclofenac to 1 µg/mL.

### 2.2 Apparatus

The experiments were conducted on an Agilent Infinite SFC system (Santa Clara, CA, USA) consisting of a SFC controlling module (G4301A), one binary pump (G4302A), one diode array detector (G1315C), an autosampler (G4303A), a degasser (G4225A), and a thermostated column compartment (G1316C). The system was controlled by an Agilent Openlab CDS Chemstation C.01.07 software. Data processing was conducted with Agilent Chemstation software. Five columns of a variety of stationary chemistries were used in the study: Waters Torus 1‐AA (1‐aminoanthrocene, 1.7 µm, 3 × 50 mm), Torus DIOL (1.7 µm, 3 × 50 mm), Torus DEA (diethylamine, 1.7 µm, 3 × 50 mm), Torus 2‐PIC (2‐picolylamine, 1.7 µm, 3 × 50 mm), and Waters HSS C18 (octadecyl bonded high strength silica) SB (1.8 µm, 3 × 50 mm).

### 2.3 Systematic study of effect of multiple injection on different stationary phases

All five columns (1‐AA, DIOL, DEA, 2‐PIC, and octadecyl bonded high strength silica) were studied in this part. The accumulative injections before the final analysis were made with neat CO_2_ as mobile phase at a flow rate of 3 mL/min. The column temperature was set at 40°C and the back‐pressure was set at 110 bar. The interval between two injections was as short as permitted by system settings (around 1 min). The mobile phase gradient after the last injection started with neat CO_2_, ramped up to 5% methanol in 0.01 min and to 25% methanol in 4.99 min, after which the mobile phase went back to neat CO_2_ for column conditioning. The flow rate of the gradient elution was 3 mL/min. The column temperature was maintained at 40°C and the back‐pressure was set at 110 bar. Both ACN and water solutions of the model compounds selected were injected. Multiple injections consisting of different numbers of single injections (1, 2, 4, 6, 8, 10, 14, and 20) were made with all standard solutions on all columns. Each multiple injection experiment was performed with three replicates. The single injection was done with a 3 µL injection loop. UV signal at 220 (for ibuprofen) and 280 nm (for other compounds) were recorded with a diode‐array detector at a sampling frequency of 20 Hz.

Within‐day precision was assessed by performing six continual multiple injection (1, 4, 8, and 12 times) of 3‐methoxycinnamic acid, sulfanilamide, ferulic acid, and p‐coumaric acid ACN solutions using the DEA column. Between‐day precision was assessed by performing multiple injection (1, 4, 8, and 12 times) of the same solutions with the DEA column in three non‐consecutive days.

The comparison experiments of one‐time large‐volume injections were carried out under the same conditions as those of the final analysis in the multiple injection experiments, except that injection loops of different volumes were used.

### 2.4 Analysis of sulfanilamide‐spiked honey extract and diclofenac‐spiked ground water

The DEA column and the 2‐PIC column were used for the analysis of sulfanilamide in honey extract and diclofenac in spiked ground water, respectively. For both analysis, the accumulative injections before the final analysis were made with 0% MeOH in CO_2_ and were terminated immediately after the final accumulative injection. Then the gradient started with neat CO_2_, ramped up to 5% in 0.01 min and to 25% in 4.99 min, held at 25% for 1 min after which the mobile phase went down to neat CO_2_. A 3 µL injection loop was used in both analysis. The column temperature was set at 45 and 40°C, and back‐pressure regulator pressure at 120 and 110 bar, respectively for the analysis of honey extract and ground water.

## 3 RESULTS AND DISCUSSION

### 3.1 Multiple injection using acetonitrile as sample diluent

For analytical SFC, acetonitrile has been proven to be a good polar sample diluent due to its aprotic feature and relatively low viscosity 14. Thus, ACN was employed as sample diluent to evaluate the performance of the multiple injection technique. In this study, the S/N enhancement of different analyte peaks varied hugely with multiple injection. Hypothetically, if an analyte band does not move or diffuse between two continual injections, the final signal enhancement ratio obtained from multiple injection compared with a single injection should be equal to the number of accumulative injections performed. However, the actual signal enhancement is highly possibly affected by three major factors: (1) Band broadening during the intervals between injections; (2) the migration of the bands during the accumulating process; (3) column overloading. There were four different scenarios observed in this work as demonstrated in Supporting Information Figure S2. Some compounds have poor retention on the column and its band moved very quickly out of the column even when the mobile phase was 100% CO_2_ during the injection intervals. Consequently, the peaks for poorly retained compounds were almost of the same size as those obtained with a single injection and the S/N enhancement ratio was around 1 (Scenario 1). For compounds that have relatively poor retention, the final peak appears split or resembles a volume‐over load wide peak (Scenario 2). This is the worst situation as there is no enhancement of S/N and the broad peak can interfere with the analysis of closely eluted adjacent peaks. Some compounds had moderate retention on the column, and the final peaks appeared to be symmetric but broadened with some gain in S/N (Scenario 3). For compounds that can be retained strongly, the final peaks presented relatively high S/N enhancement ratio, with limited peak broadening (Scenario 4). Signal to noise enhancement was assessed for all compounds until the final peak started to show shouldering or splitting after certain numbers of accumulated injections.

**Figure 2:**
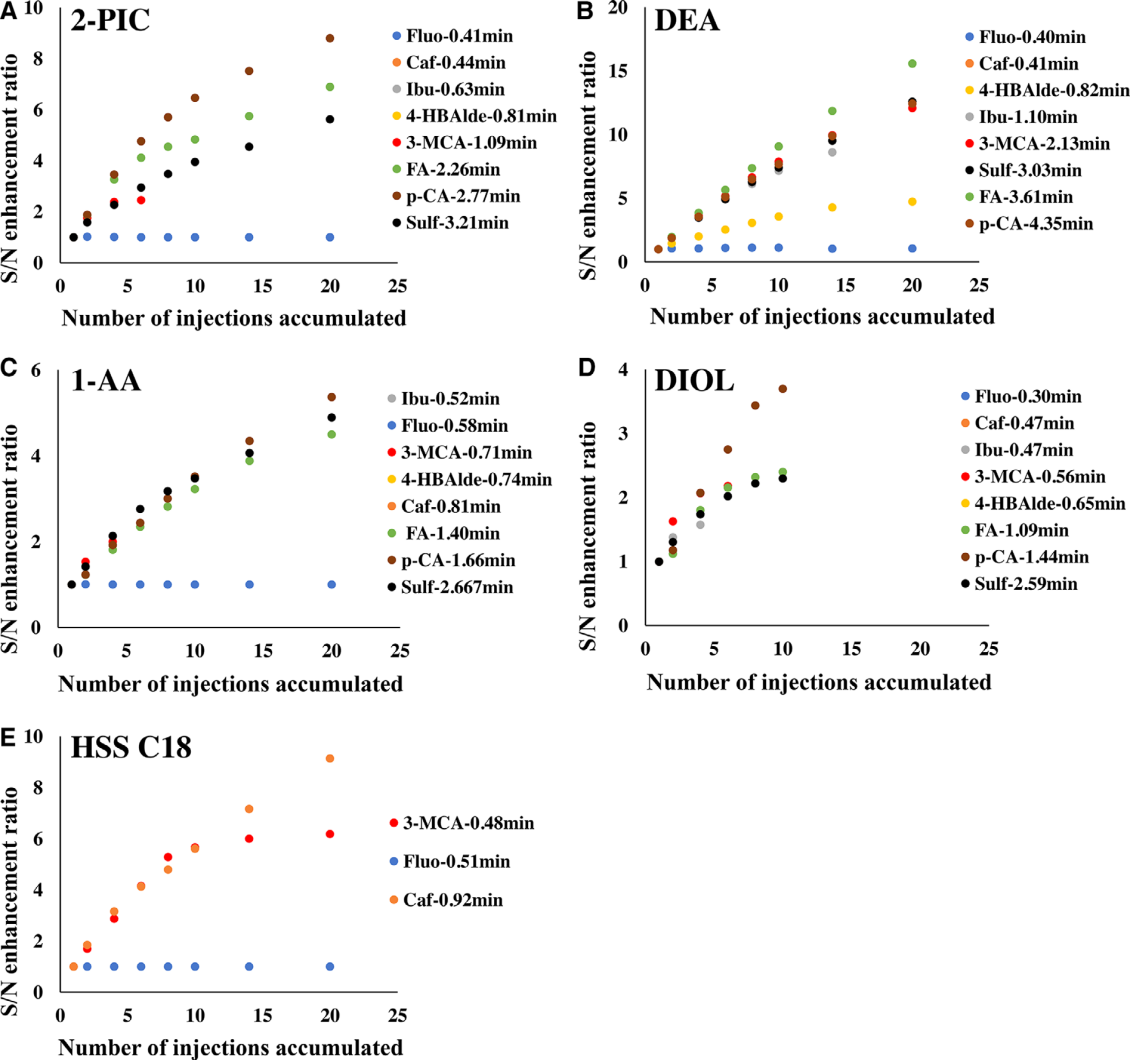
Plots of S/N enhancement ratio *vs*. number of accumulated injections with ACN as sample diluent (retention times of each compound is written besides the compound names)

As expected, multiple injection led to different S/N enhancement performances depending on the properties of the analyte and the stationary phase. Table 1 and Figure 2A show the S/N enhancement ratios of the studied analytes when a 2‐PIC column was used. In general, the longer the compound was retained, the better the S/N was enhanced. This is mainly a result from less analyte band migration during the injection intervals, as the compounds can be accumulated in an overall narrower band. However, p‐coumaric acid exhibited the best S/N enhancement as compared to sulfanilamide, although the latter had the longest retention time. This might be the result of faster migration of sulfanilamide bands under 100% CO_2_ mobile phase flow. As the mobile phase during the accumulation process did not contain any protic co‐solvent, most of the sulfanilamide molecules remained in neutral form, which could minimize the ionic interactions between the molecule and the stationary phase. In contrast, p‐coumaric acid could interact strongly with the amine moieties on the 2‐PIC stationary phase regardless of the composition of the mobile phase. Although both ibuprofen and 4‐hydroxybenzaldehyde showed poor S/N enhancement with multiple injection, the final peak of ibuprofen did not start to display shouldering until six injections had been accumulated. In contrast, 4‐hydroxybenzaldehyde peak split already after two accumulated injections, despite that 4‐hydroxybenzaldehyde had slightly longer retention time than ibuprofen. It can also be observed that the enhancement curve for some relatively more retained compounds started to show the tendency to level out after certain numbers of injections. This is the consequence of mass overloading as more and more analytes are accumulated on the column head. With the active sites at the very beginning part of the column occupied by the accumulated compounds, the later‐eluting compounds were flooded forward to bind to free active sites. Consequently, the molecules spread more widely in the column with shorted final retention time, which can be clearly observed in Scenario 4 in Supporting Information Figure S2.

**Table 1:** S/N enhancement ratios compared with single injection on a 2‐PIC column (ACN as sample diluent)

Compound	Fluo	Caf	Ibu	4‐HBAlde	3‐MCA	FA	p‐CA	Sulf
Retention time/min^a^	0.414	0.437	0.629	0.809	1.087	2.256	2.774	3.207
Single injection	1.0	1.0	1.0	1.0	1.0	1.0	1.0	1.0
2‐time injection	1.0	Split	1.8	Shoulder	1.8	1.9	1.9	1.6
4‐time injection	1.0	‐	2.3	‐	2.4	3.3	3.5	2.3
6‐time injection	1.0	‐	Shoulder	‐	2.5	4.1	4.8	2.9
8‐time injection	1.0	‐	‐	‐	Shoulder	4.5	5.7	3.5
10‐time injection	1.0	‐	‐	‐	‐	4.8	6.5	3.9
14‐time injection	1.0	‐	‐	‐	‐	5.7	7.5	4.5
20‐time injection	1.0	‐	‐	‐	‐	6.9	8.8	5.6

Fluo, fluoranthene; Caf, caffeine; Ibu, ibuprofen; 4‐HBAlde, 4‐hydroxybenzaldehyde; 3‐MCA, 3‐methoxycinnamic acid; FA, ferulic acid; p‐CA, p‐coumaric acid; Sulf, sulfanilamide.

a Retention times for single injection analysis.

Besides the properties of the compounds, the stationary phase chemistry also plays an important role in degree of enhancement of the S/N. As can be seen in Figure 2, S/N enhancement varies greatly on different columns. As had been discussed above, S/N enhancement by multiple injection generally works better for compounds that elute relatively late. The DEA column provided the best overall S/N enhancement. The S/N values increased around 10 times compared with a single injection for sulfanilamide, ferulic acid, 3‐methoxycinnamic acid, and p‐coumaric acid after 14 injections had been accumulated. In contrast, the 1‐AA and DIOL columns did not retain the compounds as strongly as the DEA column. Consequently, the S/N enhancement performances were worse with early eluting compound, i.e. peaks already split after very few accumulated injections. To further confirm the dependence of S/N enhancement on analyte retention, the retention times of all 8 compounds studied on the four Torus columns (DEA, 2‐PIC, 1‐AA, and DIOL) are displayed in Supporting Information Figure S3. Combined with the observation in Figure 2, a strong general correlation between how well the compounds are retained and S/N enhancement through multiple injection can be established.

Figure 2E shows the signal enhancement on the C18 column. Only fluoranthene, caffeine, and 3‐methoxycinnamic acid were plotted as the other compounds appeared as shouldering or split peaks even with a single injection. Multiple injection only provided moderate S/N enhancement of caffeine. Interestingly, even though fluoranthene and 3‐methoxycinnamic acid were both weakly retained with similar retention time, S/N enhancement from multiple injection was significantly better with 3‐methoxycinnamic acid than fluoranthene. This might be caused by neat CO_2_ having difference elution strength for the two compounds. During the accumulative injections with only neat CO_2_ as the mobile phase, fluoranthene is likely less adsorbed to the stationary phase and partitions more to the CO_2_ as compared to 3‐methoxycinnamic acid, since fluoranthene presents only a ring structure with no polar moiety.

**Figure 3:**
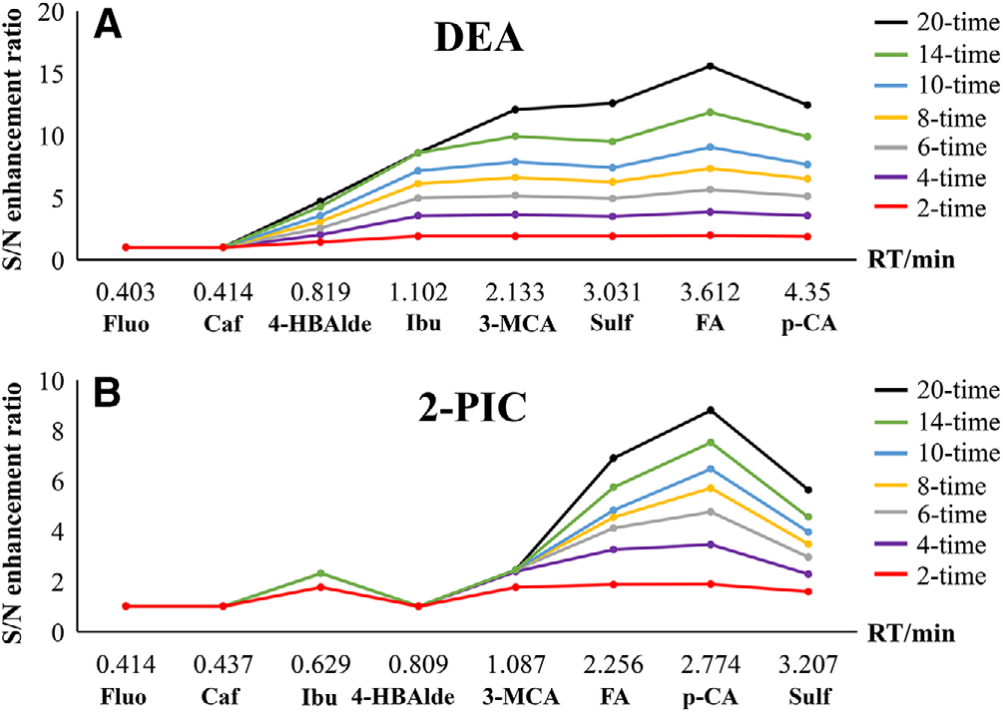
S/N enhancement and compound retention on the DEA and the 2‐PIC columns for different number of accumulated injections

As the DEA column provided the overall best signal enhancement with no peak shouldering or splitting of any compound, the S/N enhancement ratio of each compound was plotted against their retention times to illustrate the correlation. As can be seen in Figure 3A, even though the dependence of S/N enhancement on the compound retention is obvious, there seems to be a retention time threshold between 0.8 and 1.1 min after which the S/N enhancement ratio does not increase much with retention time. Similar trend was observed also with the 2‐PIC column (Figure 3B), the retention time threshold on this column appeared to be between 1.1 and 2.2 min.

### 3.2 Multiple injection using water as sample diluent

**Figure 4:**
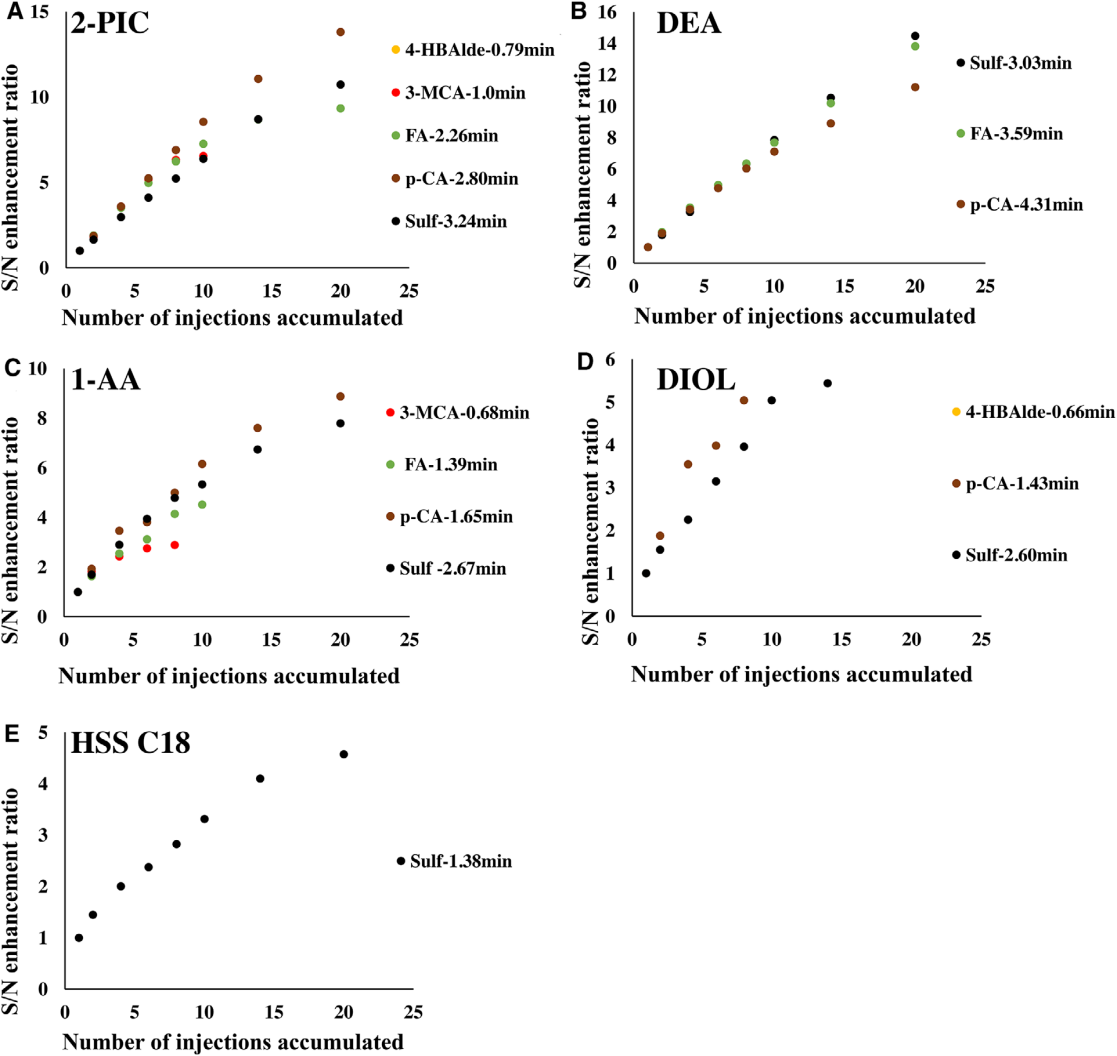
Plots of S/N enhancement ratio versus number of accumulated injections on different columns with H_2_O as a sample diluent (retention times of each compound is written besides the compound names)

The use of water has long been regarded as detrimental to SFC analysis. However, some recent studies have proven the possibility and beneficial effects of water as sample diluent 22, 23, 24, 25, 26. Water was also investigated in this work as sample diluent with multiple injection as compared to acetonitrile. Fluoranthene and ibuprofen were excluded because of solubility issues. As expected, peak shouldering or splitting occurred already with single injection for certain compounds on all columns. As the accumulation was achieved with 0% co‐solvent in the mobile phase, the huge viscosity difference between the water injection plug and the mobile phase can cause severe viscous fingering. Also, the poor miscibility of water and CO_2_ could contribute to the peak distortion as well. For the peaks which had a good peak shape with single injections, multiple injection experiments led to very different S/N enhancement (Figure 4). Also here the 2‐PIC and DEA columns provided apparently better enhancement than the other three columns. The 2‐PIC column had the best overall performance, taking into consideration the number of peaks that did not split with single injection. Interestingly, it was observed that multiple injection led to apparently increased S/N enhancement for the late eluting compounds on the 2‐PIC column when the sample diluent was changed from ACN to water, despite very little change in retention time of the compounds. The main cause of this could be that water demonstrates different solvent effects for analytes as compared to ACN and it might also change the properties of the analytes. For example, sulfanilamide can be in charged form when dissolved in water and interacts strongly with the stationary phase through ionic interaction, which is not the case with ACN as the sample diluent. Furthermore, the poor miscibility of water with neat CO_2_ enables the analyte to be injected in a relatively narrower plug compared to that with ACN as sample diluent. This demonstrates that the property of sample diluent also plays an important role in determining how well the signal is enhanced through multiple injection.

Based on all the aforementioned experimental findings, our hypothesis is that the concentrating effect of analyte on the column head through multiple injection is dependent on three major factors: compound retention, elution strength of neat CO_2_ and the type of sample diluent used.

### 3.3 Comparison with large‐volume single injection

Two comparatively late eluting compounds, p‐coumaric acid and sulfanilamide, were selected to perform large‐volume single injections as compared to the multiple injection approach. Large‐volume single injections of p‐coumaric acid and sulfanilamide standards in both ACN and water were conducted with the DEA column.

As can be seen in Table 2, similar S/N enhancement ratios were obtained for p‐coumaric acid with both injection approaches, regardless of the sample diluent. However, the advantage of multiple injection over large‐volume single injection was observed with sulfanilamide. With similar total injection volumes, multiple injection provided 2 to 3 times higher S/N enhancement than those of large‐volume single injection. The advantage of multiple injection over large‐volume single injection might be the result of different sample diluent plug dilution. Sample diluent plugs experience dilution by the mobile phase following the plug during the injection process, which can decrease its strong solvent effect and improve the concentrating effect of analyte molecules on the column head 29. Even though the dilution can happen fast, significant band broadening can take place when the injection volume is large enough. However, when this large volume injection was divided into several continual injections of a much smaller volume, each small sample diluent plug could be much more sufficiently diluted by the mobile phase during each injection than one intact large volume plug. Consequently, they displayed lower solvent effect when passing through the analyte bands. Thus, the accumulated analyte bands possibly migrated more slowly and spread less in a multiple injection process than in a large‐volume single injection. Further experiments are of course needed to validate this hypothesis. Experiments with one‐time 40 µL injection of aqueous solutions were not feasible, as a huge pressure spike took place right after the injection and quickly surpassed the upper pressure limit of the instrument, which caused system shut‐down. This phenomenon has been reported and attributed to the high pressure needed to push the viscous water sample diluent plug that is not readily soluble in the mobile phase through the column under the SFC flow rates 26. Further experiments with the 2‐PIC column showed that apparent pressure spike occurred already with 20 µL single injection of aqueous solutions (data not shown).

**Table 2:** S/N enhancement ratios for large‐volume single injection as compared to multiple injections

**ACN** as sample diluent				
	Single 20 µL injection	Multiple 3 µL × 6 injection	Single 40 µL injection	Multiple 3 µL × 14 injection
Sulfanilamide	1.93	4.91	3.16	9.52
p‐Coumaric acid	4.79	5.10	8.98	9.88

**H_2_O** as sample diluent				
	Single 20 µL injection	Multiple 3 µL × 6 injection	Single 40 µL injection	Multiple 3 µL × 14 injection
Sulfanilamide	1.59	4.76	Pressure spike above system limit	10.52
p‐Coumaric acid	4.50	4.77		8.89

Three microliter single injections were used.

### 3.4 Repeatability of multiple injection

The repeatability of the multiple injection approach has been evaluated with six experiments performed within the same day and three experiments during three non‐consecutive days. Single, 4‐time, 8‐time, and 12‐time injections were carried out for 3‐methoxycinnamic acid, sulfanilamide, ferulic acid, and p‐coumaric acid ACN solutions on the DEA column. The results are summarized in Table 3. In terms of retention time, multiple injection yielded comparable repeatability to those of single injection regardless of the number of accumulated injections. In terms of peak area, the within‐day RSD from multiple injection also resemble those from a single injection. Also, no obvious trend can be observed with different number of accumulated injections. Interestingly, multiple injection seemed to provide better repeatability than single injection based on the RSD values obtained on different days. Furthermore, between‐day RSD exhibited a clear descending trend with increasing number of injections accumulated.

**Table 3:** Repeatability of multiple injection with respect to retention time and peak area

	RSD of retention time (%)							
	3‐methoxycinnamic acid		Sulfanilamide		Ferulic acid		p‐Coumaric acid	
	Within‐day	Between‐day	Within‐day	Between‐day	Within‐day	Between‐day	Within‐day	Between‐day
Single 3 µL injection	0.070	1.5	0.21	0.52	0.22	0.71	0.078	0.41
4‐time 3 µL injection	0.13	1.4	0.23	0.61	0.088	0.68	0.051	0.49
8‐time 3 µL injection	0.080	1.3	0.12	0.89	0.047	0.59	0.043	0.41
12‐time 3 µL injection	0.081	1.2	0.052	1.1	0.061	0.61	0.042	0.49

	RSD of peak area (%)							
	3‐methoxycinnamic acid		Sulfanilamide		Ferulic acid		p‐Coumaric acid	
	Within‐day	Between‐day	Within‐day	Between‐day	Within‐day	Between‐day	Within‐day	Between‐day
Single 3 µL injection	0.76	8.7	3.6	3.6	2.8	8.7	0.92	9.7
4‐time 3 µL injection	0.68	6.9	2.5	2.8	1.6	7.6	1.4	8.4
8‐time 3 µL injection	0.27	5.8	0.35	2.3	0.83	5.6	0.31	6.6
12‐time 3 µL injection	2.19	3.6	2.3	1.4	3.1	3.6	1.9	4.5

### 3.5 Proof of concept—two application examples

Sulfanilamide is a sulfonamide that is used in some parts of the world for treating bee diseases caused by bacteria 30. Contamination of this type of sulfonamides in the honey product is a potential threat to human health because of toxicity and human allergy. Sensitive analytical methods are therefore needed in order to confirm its residue 31. An ACN extract of honey was spiked with sulfanilamide and was used to test the potential of the multiple injection approach.

**Figure 5:**
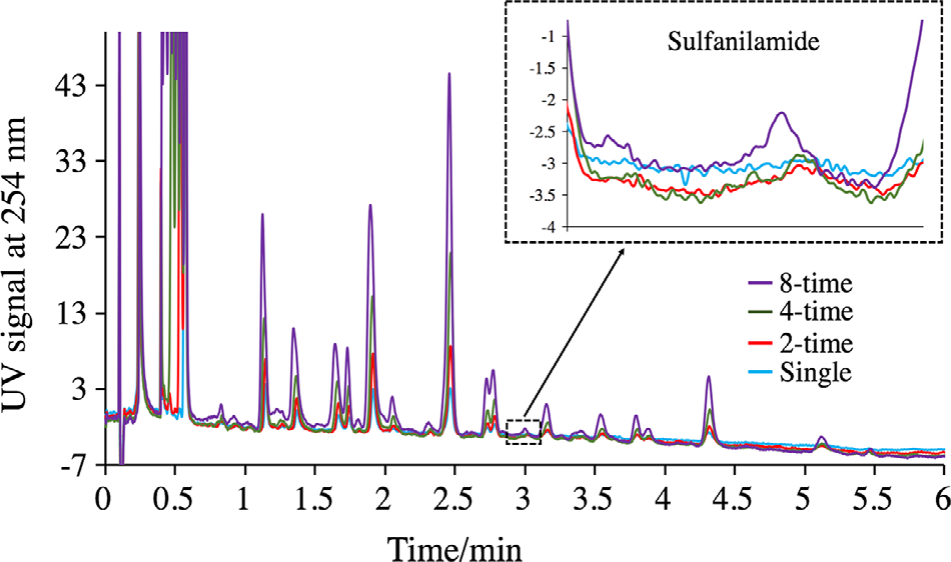
Multiple injection of sulfanilamide spiked (500 ng/mL) honey extract in ACN

Based on the S/N enhancement results of different columns in the previous sections, the DEA column was selected to perform the test. As can be seen in Figure 5, a single injection of 3 µL did not provide any recognizable peak. While more and more injections were accumulated, the sulfanilamide peak started to emerge and gradually became apparent. In contrast, the one‐time injection of a large‐volume of sample did not give the same enhancement. The repeatability of analysis of sulfanilamide in honey extract using multiple injection approach was also evaluated. Eight‐time multiple injection of honey ACN extract spiked with 2 µg/mL sulfanilamide was performed six times successively in one day and on three non‐consecutive days. The intraday and interday peak areas have RSD values of 3.6 and 7.7%, respectively. The intraday and interday retention time have RSD values of 0.83 and 1.2%, respectively.

Diclofenac belongs to the category of nonsteroidal anti‐inflammatory drugs and is widely used for treating inflammation and alleviating pain 32. As the public concern of pharmaceutical contamination in the environment is growing, studies have been made to investigate the influence of diclofenac on living creatures and have proven its harmful effects 33. For the determination of diclofenac in environmental samples that may be present in very low concentrations, an analytical method with high detectability is a necessity. In this study, a ground water sample spiked with diclofenac (1 µg/mL) was used to further prove the usefulness of the multiple injection approach.

**Figure 6:**
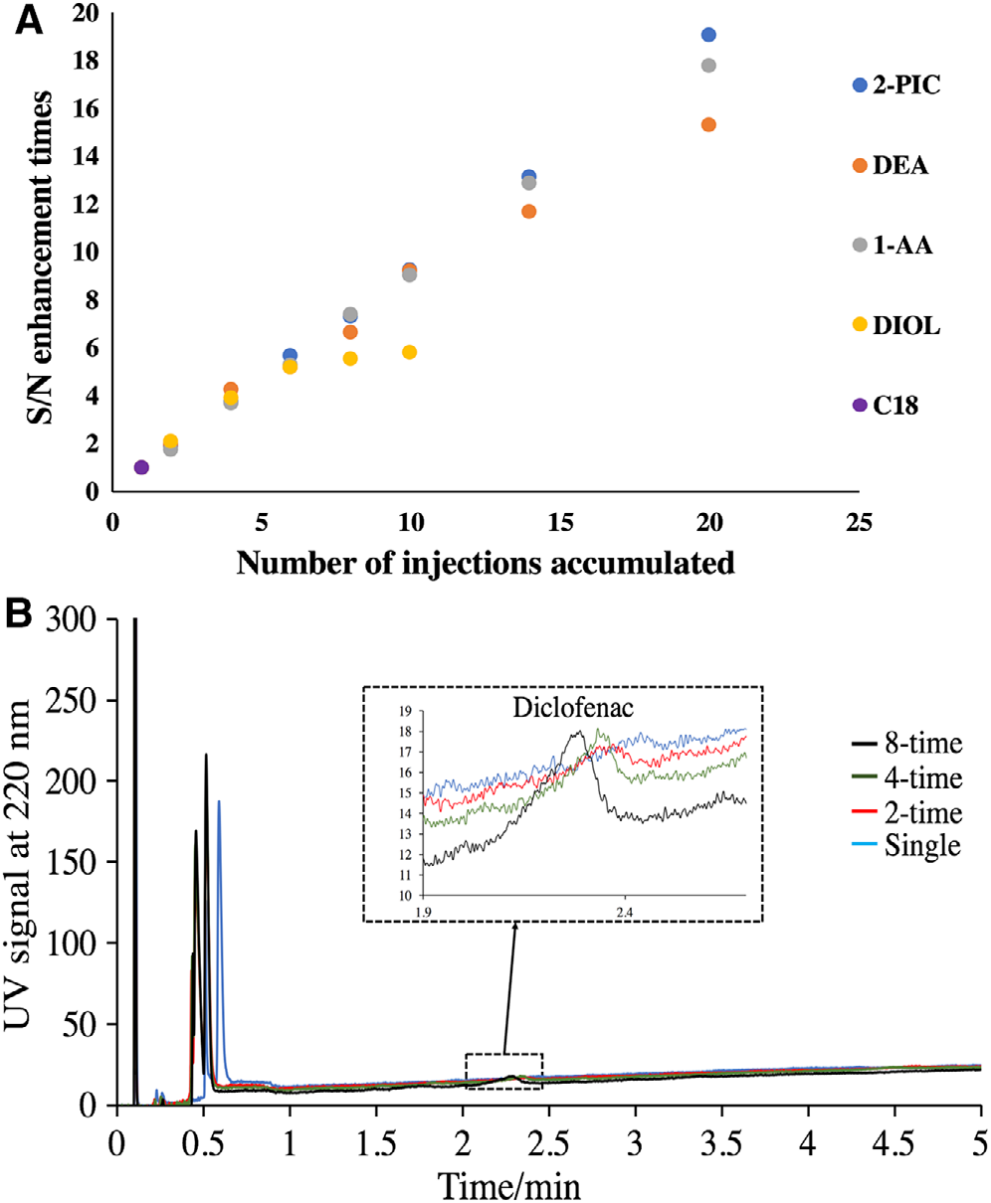
(A) S/N enhancement of diclofenac in ground water on different columns; (B) Multiple injection of diclofenac spiked (1 µg/mL) ground water sample on the 2‐PIC column

The same five columns screened in Section 3.2 were compared in terms of their S/N enhancement of diclofenac in ground water, which is depicted in Figure 6A. Peak shouldering happened after two injections accumulated on the C18 column and 10 injections accumulated on the DIOL column. The other three columns provided similarly good S/N enhancement. 2‐PIC was chosen to perform the multiple injection of spiked ground water sample, considering that it had the highest S/N enhancement ratio and the least severe peak shouldering and splitting as described in Section 3.2.

Figure 6B displays the S/N enhancement of diclofenac on the 2‐PIC column. The diclofenac peak from the single injection of 3 µL of the water sample was hardly visible, while later the peak became identifiable and even quantifiable with S/N enhancement from multiple injection. As aforementioned, one‐time injection of large‐volume water sample led to a pressure spike and noisy baseline in the chromatogram.

### 3.6 Limitations and future aspect

Even though the multiple injection technique has been proven to improve the detectability of compounds in analytical SFC when fixed‐loop injection is used, it is unavoidably associated with certain apparent limitations. First of all, relatively strong retention of the targeted analytes is a key factor to consider during column selection, which does not necessarily lead to the best resolution of the various compounds in the sample. Also, in an SFC chromatogram of a complex sample, the analyte peak of interest can be closely surrounded by adjacent peaks. Consequently, the broadening of these peaks during the accumulation process can diminish the resolution and cause difficulty in quantification. In such cases, a more selective detector like a mass spectrometer should be used.

As this work aims at demonstrating the potential and usefulness of multiple injection in modern analytical SFC, detailed optimisation of various parameters was not performed. In future application of this technique, parameters such as sample diluent, column temperature, injection volume, and number of injections should be optimised. Due to system equilibration and the needle wash pre‐programmed in the software before every injection, the interval between the injections was approximately one minute which could not be altered. This could unavoidably cause peak broadening for the relatively less retained compounds. If this interval could be freely programmed, it would be one important parameter to optimize, which could potentially improve the signal enhancement even more.

## 4 CONCLUDING REMARKS

In modern analytical SFC with fixed‐loop injection, the pursuit of higher detectability by increasing the injection volume is often associated with poor chromatographic performance. This issue can be partially circumvented by the use of a multiple injection technique as demonstrated in this work. In general, the S/N enhancement was dependent on the retention time of the compounds and the type of sample diluent. The DEA column showed the best S/N enhancement of various analytes with ACN as sample diluent. The 2‐PIC column provided the best overall S/N enhancement with water as sample diluent, considering also peak shape. The advantage of multiple injection over one‐time large‐volume single injection was proven with sulfanilamide. With similar total injection volumes, multiple injection provided two to three times higher S/N enhancement than those of large‐volume single injection. Multiple injection yielded similar repeatability in terms of retention time and peak area in comparison to those of single injection—regardless of the number of accumulated injections. The usefulness of the multiple injection approach was further demonstrated in the analysis of sulfanilamide‐spiked honey ACN extract and diclofenac spiked ground water sample.

## CONFLICT OF INTEREST

The authors have declared no conflict of interest.

## Supporting information

Supporting information
