# Supplementary material for: Signal enhancement in supercritical fluid chromatography‐diode‐array detection with multiple injection
Source: J Sep Sci. 2019 Nov 19;42(24):3727–37. doi: 10.1002/jssc.201900614 (PMC6972688; doi:10.1002/jssc.201900614)
Supplement: Supplementary file 1 — Supporting information [file JSSC-42-3727-s001.docx]

Supplementary materials to

“Signal Enhancement in Supercritical Fluid Chromatography-Diode-array Detection with Multiple Injection”

Mingzhe Sun^1^, Charlotta Turner^1^, Margareta Sandahl^1^*

*^1^ Lund University, Department of Chemistry, Centre for Analysis and Synthesis, P.O. Box 124, SE-22100 Lund, Sweden.*

**Running title***:* Signal enhancement with multiple injection in supercritical fluid chromatography

***Correspondence to**: Dr. Margareta Sandahl, Department of Chemistry, Lund University, 22100 Lund, Sweden.

**E-mail**: margareta.sandahl@chem.lu.se

**Postal Address**: P.O. Box 124 SE-221 00 Lund, Sweden.

**Article related abbreviations:**

1-AA, 1-aminoanthrocene; 2-PIC, 2-picolylamine; BPR, back-pressure regulator;

DAD, diode-array detector; DEA, diethylamine; HSS C18, octadecyl bonded high strength silica; NSAID, nonsteroidal anti-inflammatory drugs; RPLC, reversed-phase liquid chromatography; SFC, supercritical fluid chromatography

**Keywords:** Large injection volume, Multiple injection, Signal enhancement, Supercritical fluid chromatography.





Figure S1. Scheme of SFC multiple injection approach.





Figure S2. Different scenarios of S/N enhancement with multiple injection (based on data obtained with different columns in this study).


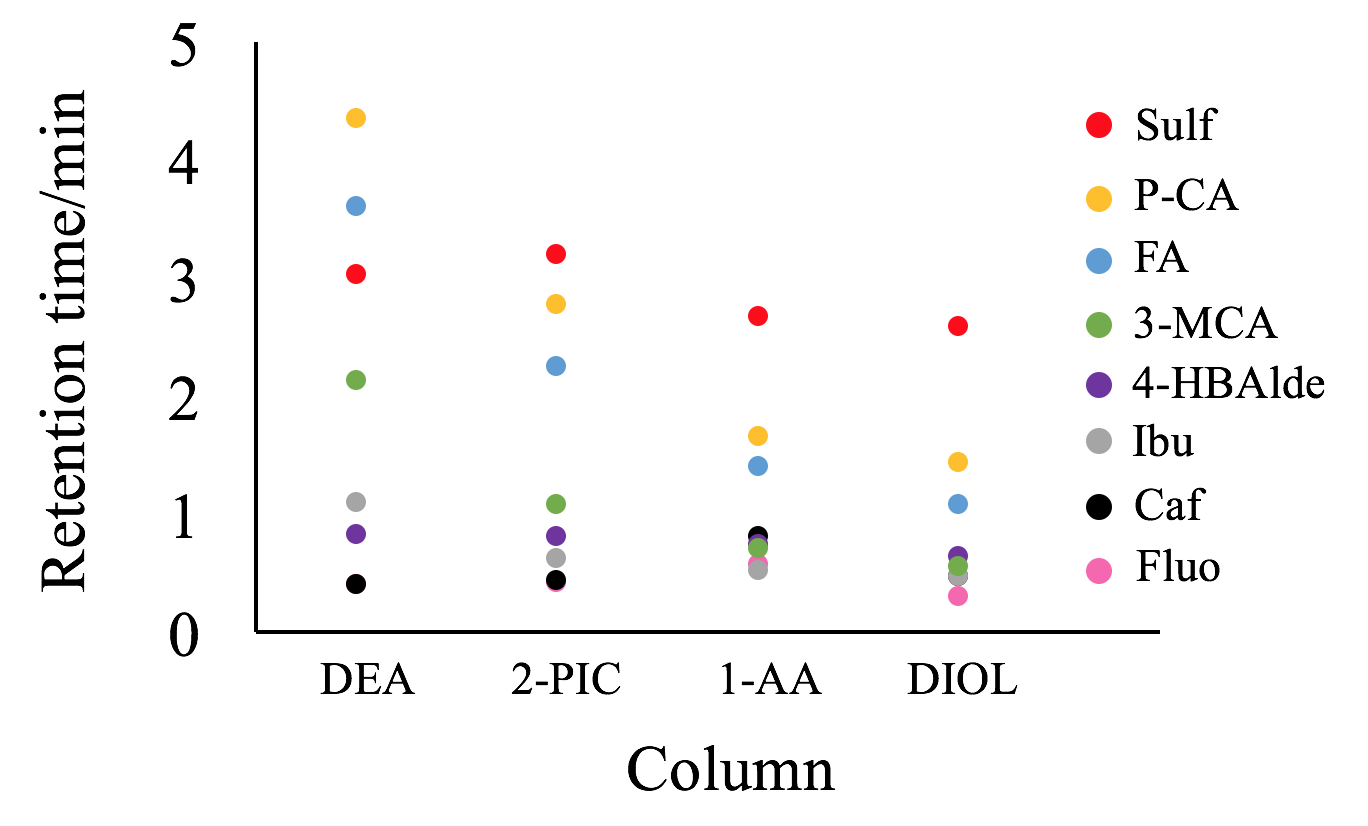


Figure S3. Compound retention time on DEA, 2-PIC, 1-AA and DIOL columns.

Table S1. Molecular structures of selected analytes

| Caffeine | Fluoranthene | Ibuprofen | p-Hydroxy benzaldehyde | p-Coumaric acid |
| --- | --- | --- | --- | --- |
|  |  |  | 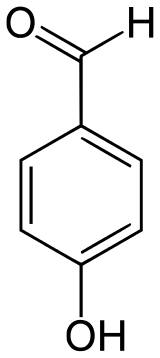 | 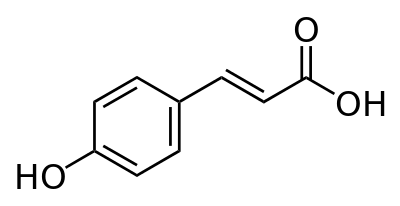 |
| 3-Methoxy cinnamic acid | Ferulic acid | Sulfanilamide | Diclofenac |  |
| 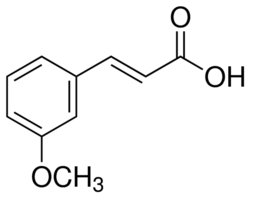 | 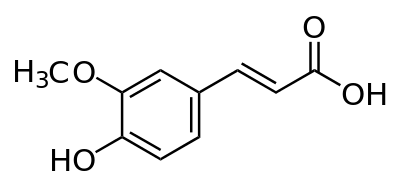 |  |  |  |
